# Thoracic Malignancies and Pulmonary Nodules in Patients under Evaluation for Transcatheter Aortic Valve Implantation (TAVI): Incidence, Follow Up and Possible Impact on Treatment Decision

**DOI:** 10.1371/journal.pone.0155398

**Published:** 2016-05-12

**Authors:** Lars Henning Schmidt, Benedikt Vietmeier, Gerrit Kaleschke, Christoph Schülke, Dennis Görlich, Christoph Schliemann, Torsten Kessler, Arik Bernard Schulze, Boris Buerke, Andreas Kuemmel, Michael Thrull, Rainer Wiewrodt, Helmut Baumgartner, Wolfgang E. Berdel, Michael Mohr

**Affiliations:** 1 Department of Medicine A, Hematology, Oncology and Pneumology, University Hospital Münster, Münster, Germany; 2 Department of Cardiovascular Medicine, Division of Adult Congenital and Valvular Heart Disease, University Hospital Münster, Münster, Germany; 3 Department of Clinical Radiology, University Hospital of Münster, Münster, Germany; 4 Institute of Biostatistics and Clinical Research, Westfälische Wilhelms-Universität Münster, Münster, Germany; 5 Pulmonary Division, Department of Medicine III, Johannes Gutenberg University Medical Center, Mainz, Germany; West German Cancer Center, GERMANY

## Abstract

**Background:**

Transcatheter aortic valve implantation (TAVI) has become the treatment of choice in patients with severe aortic valve stenosis who are not eligible for operative replacement and an alternative for those with high surgical risk. Due to high age and smoking history in a high proportion of TAVI patients, suspicious findings are frequently observed in pre-procedural chest computer tomography (CCT).

**Methods:**

CCT scans of 484 consecutive patients undergoing TAVI were evaluated for incidentally discovered solitary pulmonary nodules (SPN).

**Results:**

In the entire study population, SPN ≥ 5 mm were found in 87 patients (18%). These patients were compared to 150 patients who were incidentally collected from the 397 patients without SPN or with SPN < 5 mm (control group). After a median follow-up of 455 days, lung cancer was diagnosed in only two patients. Neither SPN ≥ 5 mm (p = 0.579) nor SPN > 8 mm (p = 0.328) were significant predictors of overall survival.

**Conclusions:**

Despite the high prevalence of SPNs in this single center TAVI cohort lung cancer incidence at midterm follow-up seems to be low. Thus, aggressive diagnostic approaches for incidentally discovered SPN during TAVI evaluation should not delay the treatment of aortic stenosis. Unless advanced thoracic malignancy is obvious, the well documented reduction of morbidity and mortality by TAVI outweighs potentially harmful delays regarding further diagnostics. Standard guideline-approved procedure for SPN can be safely performed after TAVI.

## Introduction

Lung cancer remains one of the world’s most common and most lethal cancer types [1]. To reduce incidence and mortality rates of lung cancer, both improved smoking prevention programs and early clarification of suspicious radiologic findings are essential. To improve lung cancer screening and prevention, the National Lung Screening Trial (NLST) Research Team evaluated, whether low-dose computed tomography based screening programs for patients at risk can reduce lung cancer mortality [2]. With regard to this evaluation, computer tomography based screening can be recommended for well-defined subgroups, such as heavy smokers in between 55 to 74 years [3]. Compared to colorectal cancer screening for example, improved prognostic effects of mass screening might therefore not be indicated for patients lacking specific risk constellations. Moreover, the most efficient diagnostic approach for routine screening is also not clarified, yet. One major clinical problem of computed tomography based screening is related to a high sensitivity but a rather low specificity of newly detected SPN to indicate malignant tumors [4].

Among suspicious radiologic findings especially solitary pulmonary nodules (SPN), lymphadenopathy (LAP) and pleural effusions (PE) are of clinical relevance. With regard to the size, pulmonary lesions with a diameter of less than 3 cm and which are completely surrounded by parenchyma are defined as SPN, whereas the term “tumor mass” refers to lesions above 3 cm in size [5,6]. The differential diagnosis includes both benign lesions (*e*.*g*. hamartoma or granuloma [5,7]) and malignant primary tumors [6] or secondary tumors [8]. Often, SPN are found incidentally on routine chest X-rays. One study (n = 25.529 patients above 35 years) found a prevalence of SPN of 2% in chest X-rays [9]. In this study the number of incidentally discovered SPN on CT scans was even higher with 17% [9]. Published incidence rates for incidentally discovered SPN on chest CT scans and the number of diagnosed lung cancer cases are summarized in Table 1.

**Table 1:** The incidence of incidentally discovered SPN (cases on chest computer tomographies) and the number of diagnosed lung cancer cases.

Author	Study patients (n)	Age (years)	Study population	SPN (%)	Lung cancer (%)
**Onuma et al. 2006 [**34**]**	503	66	Patients with suspected coronary artery disease	16.7	0.8
**Haller et al. 2006 [**39**]**	166	64	Patients with suspected coronary artery disease	n.e.*	1.2
**Müller et al. 2007 [**35**]**	259	64	Patients after coronary artery bypass grafting surgery	3.5	0.4
**Burt et al. 2008 [**36**]**	459	65	Patients with suspected coronary artery disease	28	n.e.*
**Machaalany et al. 2009 [**37**]**	966	58	Patients with suspected coronary artery disease	23**	0.3
**Gómez-Sáez et al. 2014 [**9**]**	2427	66	non-high-risk population	17	n.e.*
**Stachon et al. 2015 [**38**]**	374	80	Patients under evaluation for TAVR	4.3	n.e.*
**Schmidt et al. 2016**	484	82	Patients under evaluation for TAVR	18	0.6

*n.e. = not evaluable.

** granulomata included.

**Fig 1:**
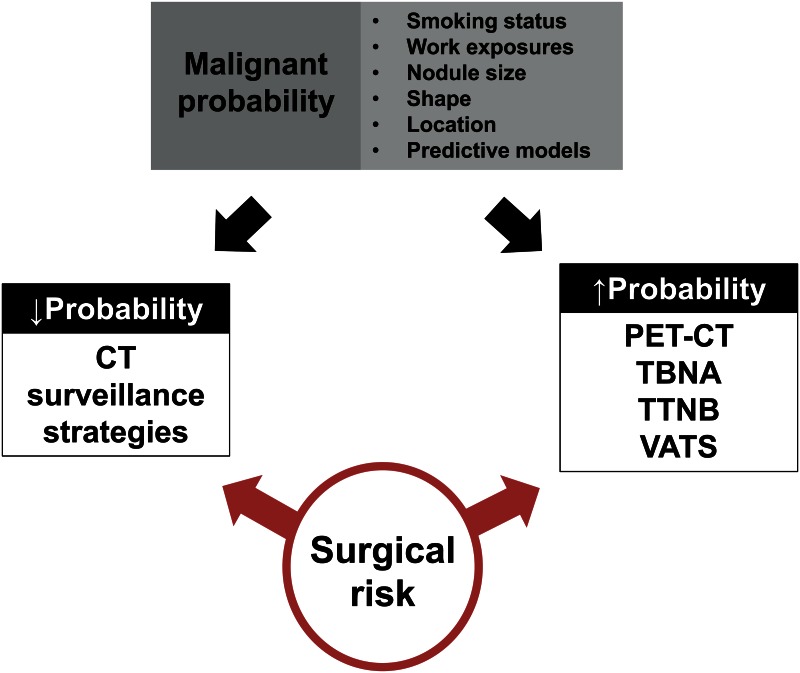
Diagnostic strategies for incidentally discovered solitary pulmonary nodules (adapted from: Gould et al. 2013).

On the basis of nodule size and risk profile (low-risk patient *vs*. high-risk patient), the Fleischner Society published guidelines for the follow-up and clinical management of incidentally discovered SPN [10]. At present, the diagnostic work-up covers positron emission tomography [11,12,13], transbronchial needle aspiration [14,15,16], transthoracic needle biopsies [17,18] and surgical intervention [19,20]. In case of low probability for malignant transformation, CT surveillance strategies are favored **[**6,10,21], with the potential risk of delayed onset of treatment (Fig 1). Of interest, chest X-rays should never be used to exclude SPN [9].

Due to rising life expectancy in Germany (http://www.destatis.de; Federal Statistical Office of Germany), incidence rates of degenerative valvular heart diseases are rising, too [22] Therapeutically, transcatheter aortic valve implantation (TAVI) has become the treatment of choice in patients with severe aortic valve stenosis who are not eligible for operative replacement [23,24]. Evaluation of the TAVI candidates is a multidisciplinary process [25]. Pre-procedural TAVI evaluation requires invasive ascending and descending aortography and or CT angiography [26]. In line with the up-coming of this technique, the number of incidentally discovered SPN in pre-procedural CT scans has risen.

Here we present single center data upon the prevalence, clinical follow-up and possible impact on therapeutic decision of incidentally discovered SPN and thoracic malignancies in patients under evaluation for transcatheter aortic valve implantation.

## Methods

### Study population

**Fig 2:**
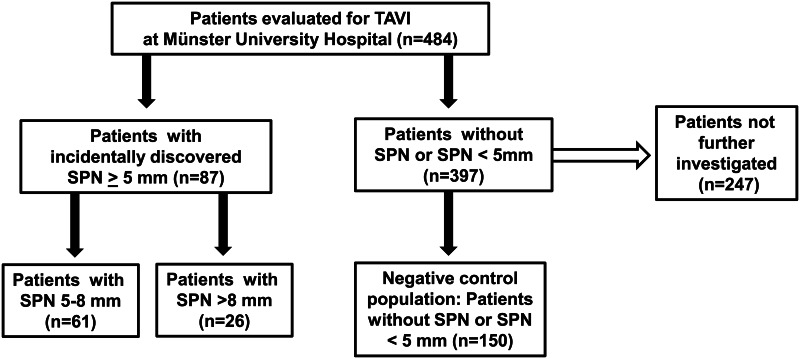
Study collective and tested clinical subgroups. The flow diagram demonstrates the selection of the tested subgroups (*control group includes both patients without detected SPN and those patients with SPN<5mm).

Before data collection, approval from the joint Ethical Committee of the Faculty of Medicine of the Westfalian Wilhelms-University Münster and the Physicians Chamber of Westfalia-Lippe was obtained (application number: 2015-037-f-S). Since the data collection was retrospective, written consent was considered as not necessary. Patient information was anonymized and de-identified prior to analysis. For all included patients, TAVI evaluation was performed at the Department of Cardiovascular Medicine, Division of Adult Congenital and Valvular Heart Disease, University Hospital Münster, Germany. To assess the overall incidence of suspicious radiologic findings in our study collective, an overall screening for solitary pulmonary nodules (SPN), lymphadenopathy (LAP) and pleural effusions (PE) was performed in 484 consecutive patients. SPN ≥ 5 mm were found in 87 patients (18%). From the remaining 397 patients without SPN or with SPN < 5 mm, 150 patients were incidentally collected for comparison (control group, Fig 2). Baseline characteristics of the two groups (*i*.*e*. patients without SPN or SPN < 4 mm *vs*. patients with SPN ≥ 5 mm) are demonstrated in Table 2. Computed power of 57.2% for both groups of patients was calculated using Statistical Analysis System (SAS Institute, Cary, NC, USA).

**Table 2:** Baseline characteristics of patients under TAVI evaluation with complete follow-up data (n = 237 patients).

	Patients with SPN ≥ 5 mm (n = 87)	Patients without SPN or SPN < 5 mm (n = 150)	p-values* for the comparison of both tested groups
**Clinical parameters**			
Median age, years (Q1–Q3)	83 (77–87)	82 (78–86)	0.571
Male gender, N(%)	37 (43%)	63 (42%)	0.937
**Respiratory parameters**			
Smoking history N(%)	26 (52%)	47 (38%)	0.096
Median FEV1% (Q1–Q3)	78% (62%-94%)	77% (64%-97%)	0.380
**Cardiologic parameters**			
LVEF, N(%)			0.148
>55%	61 (72%)	89 (60%)	
45–54%	6 (7%)	21 (14%)	
30–44%	13 (15%)	21 (14%)	
<30%	5 (6%)	17 (12%)	
Median aortic valve Area, cm² (Q1–Q3)	0.6 (0.5–0.8)	0.6 (0.5–0.8)	0.966
TAVI performed, N (%)	70 (81%)	133 (89%)	0.082
**Radiologic parameters**			
Solitary pulmonary nodule, N(%)			<0.0001
<5 mm	0 (0%)	44 (29%)	
5–8 mm	61 (70%)	0 (0%)	
>8 mm	26 (30%)	0 (0%)	
Lymphadenopathy, N(%)	18 (21%)	48 (32%)	0.061
Pleural effusions, N(%)	15 (17%)	31 (21%)	0.520
**Previous malignancy, N(%)**	20 (23%)	39 (26%)	0.605
**Lung cancer diagnosis, N (%)**	2 (3%)	0 (0%)	0.342
**Median follow-up, days** (Q1–Q3)	406 (233;603)	495 (307;859)	0.012

* p-values for the comparison of both tested groups (i.e. patients without SPN or SPN < 5 mm *vs*. patients with SPN ≥ 5 mm).

For continuous parameters Mann-Whitney-U-test and for categorical variables Chi-square test or Fisher’s exact test, respectively were applied.

### Radiological CT imaging

CT Angiography (CTA) was performed at the Department of Clinical Radiology using dual source 64 and 128 slice CT scanners (Somatom Definiton and Somatom Definition Flash, Siemens AG, Medical Solutions, Forchheim, Germany) with a tube voltage of 120 kV and a collimation of 64/128 x 0.6 mm using an attenuation-based tube current modulation (CARE dose4DTM, Siemens AG, Medical Solutions, Forchheim, Germany) to reduce radiation exposure.

Adapted to the patient’s constitution and renal function 60–100 ml i.v. contrast agent (Imeron 370, Bracco Imaging, Milano, Italy) were administered at a constant injection rate of 3–4 ml/sec. Following bolus triggered start (+140 HU, measured in the ascending aorta) images of the aortic root are acquired ECG-gated with a dose modulation, reducing the tube current between 80% and 20% of the R-R-cycle to 20%. Subsequently the whole thoraco-abdominal aorta including the inguinal arteries is acquired in one helical scan without ECG-synchronization.

All images were reconstructed in transverse orientation with a slice thickness of 1 mm and an increment of 0.6 mm for further evaluation. The ECG-gated dataset of the aortic root was additionally reconstructed in 10% steps, covering the whole R-R-cycle.

### Statistical Analysis

The study population was described by standard descriptive statistical measures. For categorical variables, absolute and relative frequencies are reported. For continuous variables median and interquartile range (IQR) are reported, respectively. To compare both tested groups (*i*.*e*. patients without SPN or SPN < 5 mm *vs*. patients with SPN ≥ 5 mm) p-values for continuous parameters were calculated using Mann-Whitney-U-test and likewise for categorical variables Chi-square test or Fisher’s exact test were applied, respectively. Similar, associations of clinico-pathological parameters with SPN were tested using two-sided Fisher’s exact test. Univariate overall survival analysis was performed using the Kaplan-Meier method and Log-rank tests. A multivariable Cox proportional hazard model was fitted using a forward step-wise variable selection (inclusion criteria: p-value of the likelihood ratio test ≤ 0.05) to identify independent prognostic factors for overall survival. We considered potential prognostic factors that are tolerably complete (less than ten missing values, and with at least ten cases), to prevent statistical problems emerging from low sample size and extreme values. Patients with missing values in the cofactors were excluded from the analysis.

All statistical tests were performed as exploratory analyses on a local significance level of 0.05. Since multiplicity adjustment was not carried out, no distinct overall significance level was ascertained. Hence, our findings may be used to set up new hypotheses. The statistical software SPSS (SPSS Statistics, Version 22.0 released 2013, IBM Corp., Armonk, NY) was used for all analyses.

## Results

### Study collectives and patient selection

Prevalence of SPN ≥ 5 mm on pre-procedural CT scans was 18% (n = 87) in the 484 patients, who were evaluated for TAVI eligibility at Münster University Hospital. Median age of the entire study population was 82 years and 202 were male (42%). Of interest, n = 111 patients (23%) had a malignant disease in the past and 3 patients were diagnosed for lung cancer following pre-procedural evaluation. All identified lung cancer patients were male smokers and had suspicious radiologic findings (*i*.*e*. patient #1: SPN of 16 mm size; patient #2: SPN of 21 mm size and lymphadenopathy; patient #3: No SPN, but tumor mass of 6.2 cm x 4 cm and lymphadenopathy). On last contact, all of them were still alive. However, TAVI was only performed on patient #1 and on patient #2.

For further diagnostic and prognostic analyses all those patients with incidentally discovered SPN ≥ 5 mm (n = 87) and n = 150 patients either without SPN or with SPN < 5 mm (serving as the control population) were chosen (computed power: 57.2%). Depending on the size of SPN, n = 61 patients had SPN of 5–8 mm and n = 26 patients had SPN of > 8 mm size. Of the identified three lung cancer patients, only two patients had SPN by definition. Even though patient #3 was also diagnosed with lung cancer, this patient was excluded from further statistical analyses due to the initial discovery of a “tumor mass” in the pre-procedural CT scan.

Baseline characteristics for both tested study collectives (in total n = 237 patients: n = 150 patients without SPN or SPN < 5 mm *vs*. n = 87 patients with SPN ≥ 5 mm) are summarized in Table 2. Regarding equal distribution of the two tested study collectives, no significant difference was found for the evaluated variables (e.g. age, gender or smoking history). Since the SPN size was command variable, a significant difference was found (p < 0.0001).

### Clinical associations for identified SPN

For the 237 study patients associations of clinical and respiratory parameters with suspicious radiologic findings (*i*.*e*. SPN ≥ 5 mm, SPN > 8 mm, LAP and PE) were investigated. Here, smoking history was positively associated with SPN > 8 mm (p = 0.033). Suspicious lymphadenopathy was found more often for patients below 80 years in contrast to patients above 80 years (39% *vs*. 23%; p = 0.013) and for men in contrast to women (36% *vs*. 22%; p = 0.019). For all other tested variables no other relevant correlations with SPN, LAP or PE were found (all p>0.05; Table 3).

**Table 3:** Correlations of clinical and respiratory parameters with suspicious radiologic findings (*i*.*e*. SPN, LAP and PE) for patients under TAVI evaluation with complete follow-up data (n = 237 patients).

	p-values according to Fisher’s exact test			
	SPN ≥ 5mm (n = 87)	SPN > 8mm (n = 26)	LAP (n = 66)	PE (n = 46)
**Clinical parameters**				
Age (< 80 years *vs*. ≥ 80 years)	0.667	0.510	0.013	0.053
Sex (male *vs*. female)	1.000	0.097	0.019	0.621
**Respiratory parameters**				
Smoking history (non-smokers *vs*. smokers)	0.126	0.033	0.741	0.414
FEV1% (FEV ≥ 80% *vs*. FEV1 < 80%)	0.741	0.318	0.727	0.401
Previous malignancy	0.643	0.632	0.180	1.000

### Univariate prognostic effects

**Fig 3:**
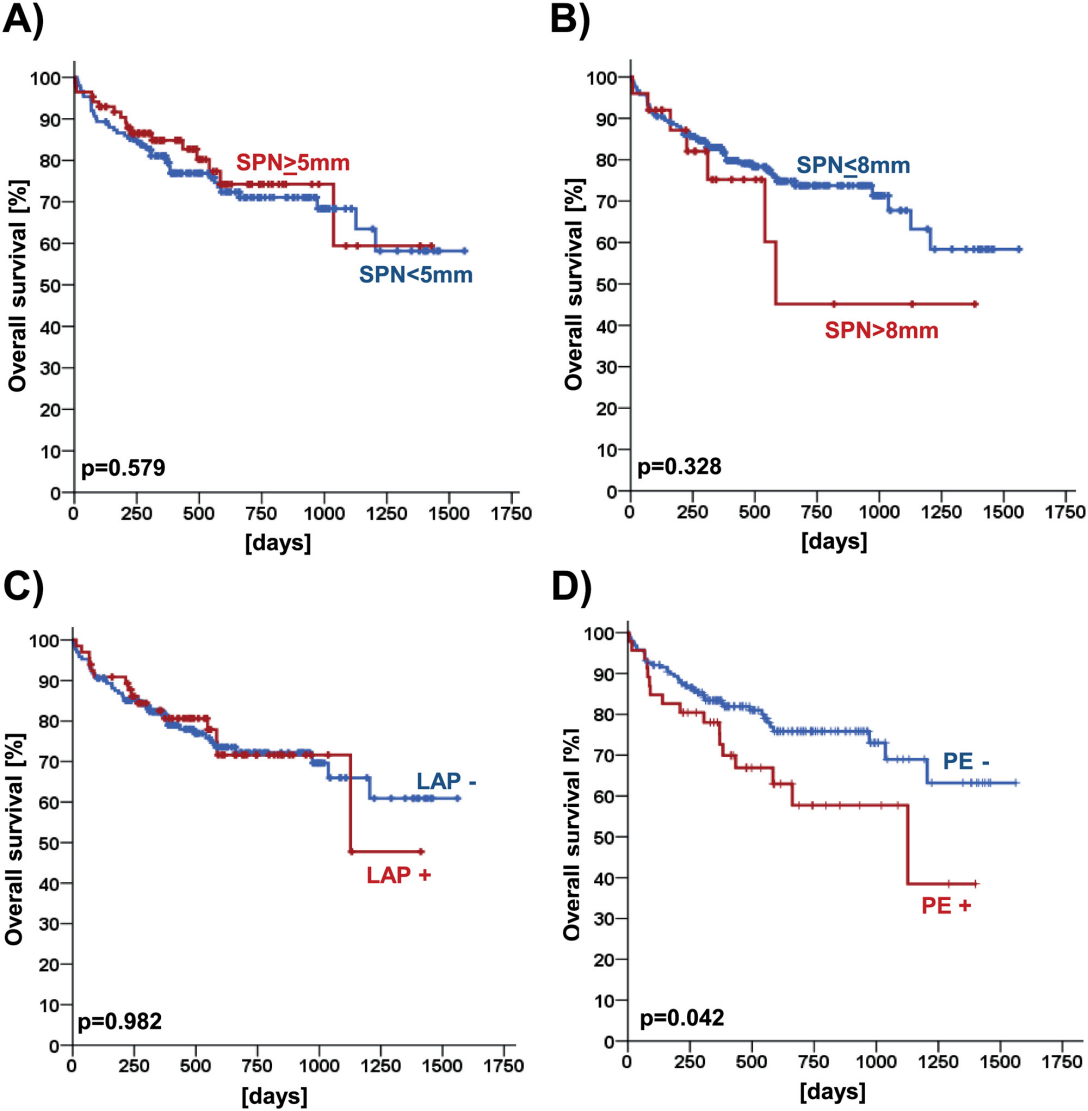
Prognostic impact of solitary pulmonary nodules (SPN), lymphadenopathy (LAP) and pleural effusions (PE) in patients under evaluation for TAVI (n = 237 patients). Kaplan Meier charts are given for SPN ≥ 5mm (**A**), for SPN > 8mm (**B**), for LAP (**C**) and for PE (**D**).

Using Log-rank test, prognostic impact of SPN ≥ 5 mm and of SPN ≥ 8 mm in patients under TAVI evaluation with complete follow-up data (n = 237 patients) was investigated. Neither for SPN ≥ 5 mm nor for SPN ≥ 8 mm prognostic effects were found in these univariate analyses (all p > 0.05, Table 4 and Fig 3). In addition, prognostic analyses were also performed for suspicious lymphadenopathy (p > 0.05; Table 4 and Fig 3) and pleural effusions (p = 0.042; Table 4 and Fig 3).

**Table 4:** Prognostic analysis for suspicious radiologic findings (*i*.*e*. SPN, LAP and PE) in patients under TAVI evaluation with complete follow-up data (n = 237 patients).

	p-value according to log rank test			
	SPN ≥ 5mm (n = 87)	SPN > 8mm (n = 26)	LAP (n = 66)	PE (n = 46)
**All patients, full study collective**	0.579	0.328	0.982	0.042
**Subgroup: Clinical parameters**				
Age				
< 80 years	0.268	0.382	0.824	0.015
≥ 80 years	0.179	0.521	0.798	0.257
Sex				
male	0.227	0.677	0.959	0.140
female	0.716	0.057	0.984	0.214
**Subgroup: Respiratory parameters**				
Smoking				
never-smokers	0.206	0.383	0.575	0.954
smokers	0.347	0.579	0.645	0.083
FEV1%				
FEV1 ≥ 80%	0.663	0.799	0.824	0.040
FEV1 < 80%	0.468	0.969	0.712	0.991
Previous malignancy				
no previous malignancy	0.299	0.560	0.987	0.035
previous malignancy	0.472	0.279	0.938	0.749

### Multivariate prognostic effects

Cox proportional hazards models for comparison with established prognostic factors was used to identify prognostic impact in a multivariate setting. Included variables were: gender (male (ref.) *vs*. female), age (< 80 years (ref.) *vs*. ≥ 80 years), left ventricular ejection fraction (both as continuous variable and as categorical variable (LVEF < 45% (ref.) *vs*. LVEF ≥ 45%)), previous malignancy (no previous malignancy (ref.) *vs*. previous malignancy), solitary pulmonary nodules (no SPN (ref.) *vs*. SPN ≥ 5 mm and all others (ref.) *vs*. SPN > 8 mm), lymphadenopathy (no LAP (ref.) *vs*. LAP) and pleural effusions (no PE (ref.) *vs*. PE).

**Fig 4:**
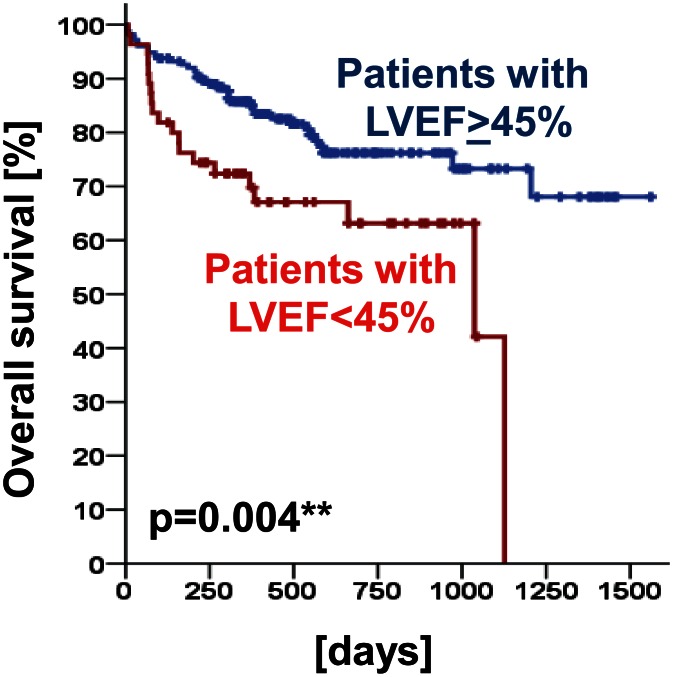
Prognostic impact of left ventricular ejection fraction (LVEF) in the full study collective (p = 0.004). Overall survival of those patients with a LVEF ≥ 45% was increased compared to those patients with a LVEF < 45%.

As shown before in the univariate analyses, SPN ≥ 5 mm or SPN > 8 mm were not identified as independent prognostic factors (p ≥ 0.05; Table 5). However, left ventricular ejection fraction was found to be of prognostic relevance for overall survival (HR [95% CI] = 2.194 [1.220–3.947]; p = 0.009; Table 5). This observation was next confirmed using log rank test (p = 0.004; Fig 4).

**Table 5:** Overall survival: Explanatory prognostic factors in a Cox proportional Hazards model for the selected study collective. Included variables: sex (male (ref.) *vs*. female), age (< 80 years (ref.) *vs*. ≥ 80 years), LVEF (as a continuous variable), LVEF (LVEF < 45% (ref.) *vs*. ≥ 45%); previous malignancy (no previous (ref.) malignancy *vs*. previous malignancy); SPN (no SPN (ref.) *vs*. SPN ≥ 5 mm and all others (ref.) *vs*. SPN > 8 mm), lymphadenopathy (no lymphadenopathy (ref.) *vs*. lymphadenopathy) and pleural effusions (no pleural effusions (ref.) *vs*. pleural effusions).

Identified prognostic factor	p-Value*	HR^1^ (95% CI)^2^
LVEF	0.009	2.194 (1.220–3.947)

^1^ HR = hazard ratio: HR <1 indicates improved survival.

^2^ CI = confidence interval.

* p value indicates model improvement (likelihood ratio test).

## Discussion

Both the diagnostic and therapeutic management of incidentally discovered SPN depends on the evaluation of malignant probability. Therefore, risk factors such as smoking habits or work-related exposures need to be considered. Next, the exact description of shape and size is important. The bigger the nodule, the higher the chance for malignant transformation [10,27–31]. Likewise, information regarding location [30], growth [32] and shape [33] should be respected, too. Predictive models such as the Mayo Clinic model (derived from chest X-rays with focus on age, smoking status, history of malignancy, SPN size, shape and location [27]) or computer-based calculation programs (e.g. http://chestx-ray.com/index.php/calculators/spn-calculator) can facilitate risk stratification.

Although the number of elderly patients who require TAVI implantation is growing [24], there are neither evidence based recommendations nor clinical guidelines to decide upon the further therapeutic procedure of incidentally discovered SPN during pre-procedural TAVI evaluation. Hence, there is a growing demand for interdisciplinary decisions on priorities for further diagnostic versus therapeutic procedures in these patients.

To address this clinical problem, we evaluated pre-procedural CT scans of 484 patients with focus on solitary pulmonary nodules (SPN). SPN of at least 5 mm size were found in 18% in the initial study collective. This ratio corresponds well to other published studies, which reported incidence rates ranging from 3.5% to 28% [9,34–39].

To investigate the prognostic impact of SPN in patients who were under investigation for TAVI eligibility, we included only those 87 patients with incidentally discovered SPN ≥ 5 mm and collected 150 control patients either without SPN or with SPN < 5 mm. For all these patients (n = 237 patients in total) complete follow-up data were assigned and clinical correlations were studied. Besides, lymphadenopathy was found in 28% and pleural effusions in 19% of the investigated patients. Upon further diagnostics, 0.6% of the patients developed lung cancer. This ratio corresponds well to other reported ratios ranging from 0.3% to 1.2% [34–35,37,39].

With regard to clinical correlations, no relevant associations were found for SPN ≥ 5. For smoking history however, a positive association with larger SPN of > 8 mm was observed. This association is also supported by another study group [9]. Besides, we found positive associations for lymphadenopathy with age and gender. To our knowledge, there are no other studies, which contribute to this observation. Potentially, this observation might only hint to a better general health status of women compared to men above the age of 80 years in our study collective. Hence, we also investigated the prognostic impact of gender on overall survival. However, for the investigated study collective we cannot report a relevant prognostic impact of gender on overall survival (data not shown).

Regarding prognosis, the incidence of SPN did not affect overall survival neither within the investigated entire study collective nor within clinical subgroups. In the univariate model, prognostic effects were found for pleural effusions in the full study collective and even stronger effects within the tested prognostic subgroups stratified for age, impaired FEV1 and a history of previous malignancies. In general, pleural effusions can be found in various clinical conditions. Besides, rheumatoid, infectious or malignant diseases [40], pleural effusions are often associated with cardiac failure [41]. Hence, the prognostic impact of pleural effusions might rather reflect cardiac failure in this population. In our study collective impaired LVEF was associated with pleural effusions (data not shown). This hypothesis can also be supported by the observed prognostic impact of LVEF both in the univariate and in the multivariate analysis.

With regard to this prognostic approach, further diagnostic procedures for incidentally discovered SPN delaying TAVI evaluation may not yield in improved prognosis for this patient collective and delay symptom amelioration and prognostic effects of TAVI. Similar to our approach, another research group focused on suspicious incidental radiological findings in 414 participants screened either for surgical aortic valve replacement or TAVI with dual-source CT scans [38]. According to their study, suspicious incidental radiological findings did not significantly influence further therapeutic decisions [odds ratio (OR) 1.14; P = 0.835] or time to treatment (91 ± 152 *vs*. 61 ± 109 days, respectively). Moreover, overall survival two years after decision to intervene did not differ significantly (p>0.05, [38]). The decision for treatment was made according to the guidelines for TAVI, which recommend the procedure only in patients with an estimated survival of 1 year at least [42]. Besides, decisions should be made up by interdisciplinary teams [42]. Our analysis indicates a need for more specific clinical recommendations or guidelines to decide on the further evaluation of patients at risk with newly detected SPN. Potentially, CT follow-up scans for patients with SPN in a good clinical status (i.e. NHYA I) might therefore be recommended after a period of six months. Otherwise, our results as well as other published results argue in favor of the notion, that standard guideline-approved procedures for SPN can be safely performed after TAVI. Besides its single-center nature, the retrospective study design and the interdisciplinary selection for TAVI intervention our study does have its limitations. However our results may be helpful in management of patients evaluated for TAVI who present with suspicious radiologic findings.

In conclusion, we were able to demonstrate, that the overall survival of those patients, who are under evaluation for TAVI was not affected by incidentally detected SPN. Thus, aggressive diagnostic approaches for incidentally discovered SPN during TAVI evaluation should not delay the treatment of aortic stenosis. Unless advanced thoracic malignancy is obvious, the well documented reduction of morbidity and mortality of severe symptomatic aortic stenosis by TAVI outweighs potentially harmful delays regarding further diagnostics. Standard guideline-approved procedure for SPN can be safely performed after TAVI.

## Supporting Information

S1 Table Initial study collective. Baseline characteristics of all patients under TAVI evaluation (n = 484 patients). (DOCX)
