# Supplementary material for: Thoracic Malignancies and Pulmonary Nodules in Patients under Evaluation for Transcatheter Aortic Valve Implantation (TAVI): Incidence, Follow Up and Possible Impact on Treatment Decision
Source: PLoS One. 2016 May 12;11(5):e0155398. doi: 10.1371/journal.pone.0155398 (PMC4865104; doi:10.1371/journal.pone.0155398)
Supplement: S1 Table — (DOCX) [file pone.0155398.s001.docx]

|  | All Patients (n=484) |
| --- | --- |
| **Clinical parameters** |  |
| Median age, years (Q1-Q3) | 82 (77-86) |
| Male gender, N(%) | 202 (42%) |
| **Respiratory parameters** |  |
| Smoking history N(%) | 73 (42%) |
| Median FEV1% (Q1-Q3) | 78% (62%-93%) |
| **Cardiologic parameters** |  |
| LVEF, N(%) |  |
| >55 % | 158 (65%) |
| 45-54 % | 28 (11%) |
| 30-44 % | 37 (15%) |
| <30 % | 22 (9%) |
| Median aortic valve Area, cm² (Q1-Q3) | 0.6 (0.5-0.8) |
| TAVI performed, N (%) | 211 (84%) |
| **Radiologic parameters** |  |
| Solitary pulmonary nodule, N(%) |  |
| <5 mm | 0 (0%) |
| 5-8 mm | 61 (13%) |
| >8 mm | 26 (5%) |
| ≥10 mm | 22 (5%) |
| Lymphadenopathy, N(%) | 121 (25%) |
| Pleural effusions, N(%) | 90 (19%) |
| **Previous malignancy, N(%)** | 111 (23%) |
| **Lung cancer diagnosis, N (%)** | 3 (1%) |
| **Median follow-up, days** (Q1-Q3) | 455 (269;740) |
